# Single or multiple pulsed field applications: Intracardiac electrogram changes and implications for procedural end points

**DOI:** 10.1016/j.hroo.2026.01.019

**Published:** 2026-01-27

**Authors:** Vincent Schlageter, Patrick Badertscher, David Spreen, Adrian Luca, Philipp Krisai, Behnam Subin, Nicolas Schaerli, David Zivanovic, Thomas Kueffer, Felix Mahfoud, Michael Kühne, Christian Sticherling, Sven Knecht

**Affiliations:** 1 Department of Cardiology, University Hospital Basel, University of Basel, Basel, Switzerland; 2 Cardiovascular Research Institute Basel, University Hospital Basel, University of Basel, Basel, Switzerland; 3 Signal Processing Laboratory, Swiss Federal Institute of Technology, Lausanne, Switzerland; 4 Department of Cardiology, Lausanne University Hospital, University of Lausanne, Lausanne, Switzerland; 5 Department of Cardiology, Inselspital, Bern University Hospital, University of Bern, Bern, Switzerland; 6 Department of Rhythmology, University Hospital Lübeck, Lübeck, Germany

**Keywords:** Pulsed field ablation, Electrogram, Acute effect, End point definition, Bipolar voltage, Pulmonary vein isolation, Frequency analysis

## Abstract

**Background:**

Pulsed field ablation (PFA) using a pentaspline catheter is increasingly applied for pulmonary vein (PV) isolation, but standardized methods to assess durable lesion formation are lacking.

**Objective:**

We aimed to investigate the impact of incremental PFA applications on electrogram (EGM) characteristics and determine whether EGM signatures serve as markers for effective lesions.

**Methods:**

PV EGMs of a cohort of consecutive patients were analyzed before and after single, double, and quadruple PFA applications. Bipolar voltage amplitude (BVA) and signal power in different frequency bands (>50 Hz, >75 Hz, and >125 Hz [HF125]) were quantified. EGMs were reassessed after 60 seconds, and pacing was performed to evaluate PV pace capture.

**Results:**

This analysis included 4230 EGMs of 83 patients. Immediately after ablation, no differences in BVA and frequency power were observed between groups. After 60 seconds, significant differences were present for all features in the single, double, and quadruple groups: BVA (0.27 mV vs 0.26 mV vs 0.13 mV), >50 Hz (39 μV^2^ vs 35 μV^2^ vs 8 μV^2^), >75 Hz (10 μV^2^ vs 13 μV^2^ vs 3 μV^2^), and HF125 (3 μV^2^ vs 2 μV^2^ vs 1 μV^2^). EGM features recovered within 60 seconds in the single and double groups but not in the quadruple group. Pace capture at 5 V was best predicted by an HF125 threshold of 2.5 μV^2^ and a BVA threshold of 0.28 mV.

**Conclusion:**

Only quadruple PFA applications ensured persistent lesion effects over the observation period. A combined end point using an HF125 cutoff of 2.5 μV^2^ and BVA cutoff of 0.28 mV may provide accurate confirmation of PV isolation with the pentaspline catheter.

## Graphical abstract

**Figure:**
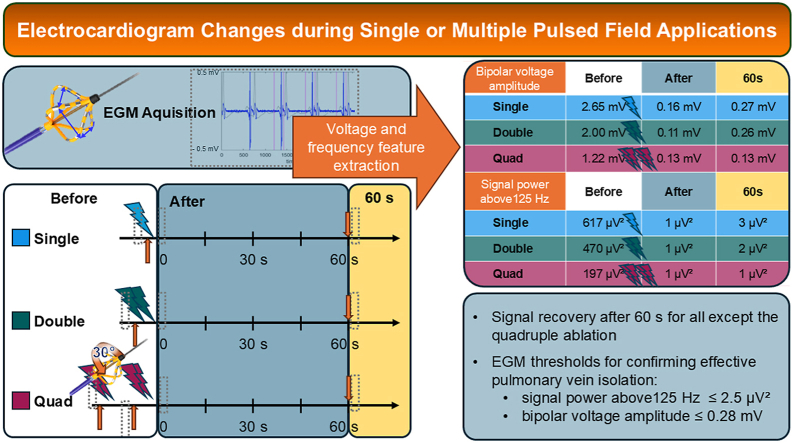

**Key Findings**

▪ Immediately after ablation, no difference in electrogram (EGM) characteristics between single, double, or quadruple pulsed field ablation (PFA) applications was observed.
▪ Over a 60-second waiting period, only the quadruple PFA applications resulted in lasting lesion effects.
▪ The specific EGM thresholds of the signal power above 125 Hz of ≤2.5 μV^2^ or bipolar voltage amplitude of ≤0.28 mV were identified as potential electrophysiological markers for confirming effective pulmonary vein isolation.

## Introduction

Pulsed field ablation (PFA) is a novel technology for catheter ablation of atrial fibrillation (AF). Potential advantages over conventional thermal ablation modalities include enhanced safety profile, reduced collateral tissue damage, and tissue-selective ablation properties.^1^ Despite fast clinical adoption of PFA, standardized criteria for real-time assessment of lesion formation remain incompletely defined.

Conventional markers of lesion formation used during radiofrequency (RF) ablation, such as reductions in bipolar electrogram (EGM) amplitude, impedance drop, and catheter tip temperature monitoring, have long served as procedural surrogates of tissue injury. However, these markers have limited applicability in the setting of PFA. PFA differs fundamentally from RF because it induces cell death through electroporation rather than thermal injury, leading to distinct acute electrophysiological signatures.^2^ Indeed, recent observations suggest that EGM changes after PFA can evolve dynamically over time, likely reflecting mechanisms of transient or reversible cellular depolarization.^3^ This raises the need to identify novel and specific EGM markers that can reliably and acutely confirm effective lesion formation in the context of electroporation-based ablation.

Despite the growing adoption of PFA, important gaps remain in our mechanistic understanding of its electrophysiological correlates. In particular, the incremental effect of stacking multiple PFA applications and the EGM features that predict durable lesion formation are not well defined. As a result, there is a pressing need for objective, electrophysiology-based markers to guide energy delivery, optimize efficiency, and avoid unnecessary applications.

To address this gap, this study systematically characterizes EGM “fingerprints” of PFA using frequency-domain analysis across incremental applications. By defining reproducible electrophysiological signatures that correlate with acute lesion formation as validated by nonexcitability by pacing, we aimed to establish potential procedural end points that may predict durable lesion success. Such findings could enable a more mechanistic approach to PFA guidance, inform standardized delivery protocols, and ultimately improve both procedural efficiency and long-term clinical outcomes.

## Methods

### Patient population

We prospectively included consecutive patients referred for catheter ablation of AF using the pentaspline PFA catheter (Farawave, Boston Scientific) between January and October 2024. Patients with previous left atrial ablation or in whom sinus rhythm could not be restored by cardioversion before the first PFA application were excluded. All patients provided a written informed consent. The study was approved by the local ethics board (Swiss Atrial Fibrillation Pulmonary Vein Isolation Registry: NCT03718364) and performed in accordance with the Declaration of Helsinki. The research reported in this paper adhered to Consolidated Standards of Reporting Trials guidelines.

### Catheter ablation

**Figure 1:**
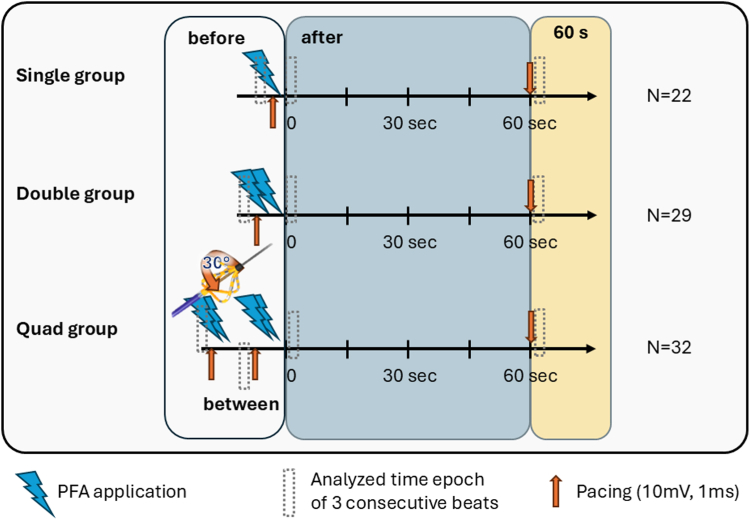
Schematic representation of the ablation protocol for the 3 groups with the number of patients included. PFA = pulsed field ablation.

The ablation was performed as recently published.^4^^,^^5^ Briefly, the PFA catheter was inserted into the left atrium after fluoroscopically guided transseptal puncture and positioned at the ostium of the pulmonary vein (PV) in basket configuration. Proper orientation and electrode contact with the myocardium were confirmed fluoroscopically by the deformation of the catheter splines. We implemented 3 different workflows: a single application (single group), a double application (double group) with 2 consecutive applications in the same location without rotating the catheter, and a quadruple application (quad group) protocol with the catheter rotated by ∼30° after the second application (Figure 1). Before continuing with further PFA application for PV isolation (PVI) as recommended by the company (overall 4 basket and 4 flower applications), a 60-second waiting period was applied in all groups to assess the temporal evolution of the EGM.

Acute lesion efficacy was evaluated using a pacing protocol to determine whether the targeted myocardial tissue had become unexcitable. Pacing was performed at 10 V with a pulse width of 1 ms and for the last 11 patients in the quad group at 5 V with a pulse width of 1 ms across all 5 bipoles. Pacing assessments were performed at 2 time points: before the first ablation application to define baseline conditions and after ablation after a 60-second waiting period during which the catheter remained in the same position. In the quad group, pacing was also performed between the 2 ablation applications after rotation of the catheter. Recovery of the signal features was calculated as the percentage change at 60 seconds relative to the feature values immediately after ablation (after).

### Signal processing and EGM feature extraction

Intracardiac bipolar EGMs of the PFA catheter of all PVs were sampled at 2000 Hz and processed using a bandpass filter with cutoff frequencies at 30 Hz and 300 Hz, along with a 50 Hz notch filter to suppress power line interference. EGMs with excessive noise, as determined by assessment of a reference period preceding the P wave, were excluded.

For each of the 5 bipolar EGMs of the PFA catheter, the bipolar voltage amplitude (BVA) and the absolute power in the high-frequency (HF) range (20–150 Hz) were extracted for 3 consecutive beats for each epoch using a 60 ms sliding window and keeping the maximum value. The EGM feature of HF was calculated via fast Fourier transform, using zero padding to achieve a frequency resolution of 5 Hz, for near-field signal discrimination.^6^ The extracted HF power values were reported using a high-pass filter of 50 Hz, 75 Hz, and 125 Hz. All features were computed for 3 consecutive beats, and the median value was retained for further analysis.

### End points definition

The presence or absence of pacing capture was reported by the operating physician for each of the 5 electrode pairs based on a stable local atrial capture for 3 consecutive beats. This binary outcome was used as ground truth to assess acute lesion effectiveness. Each signal feature (BVA and HF) was independently used to predict pacing capture, and balanced accuracy (BA) was calculated to evaluate predictive performance.

### Statistical analysis

Continuous variables are presented as mean ± standard deviation or as median (interquartile range [IQR]) as appropriate. Analysis of variance with post hoc Bonferroni-corrected pairwise comparison was performed to test for the effect over time for all features separately. Differences in continuous variables across the groups were analyzed using 1-way analysis of variance. Comparison of the prevalence of categorical factors among the groups was performed using Pearson’s χ^2^ test or Fisher’s exact test where appropriate. Model performance to predict pace capture was evaluated using a confusion matrix, and sensitivity (true positive rate), specificity (true negative rate), precision (positive predictive value), and BA were calculated for HF range between 20 and 150 Hz in steps of 5 Hz. Analyses were performed using MATLAB (MathWorks, Inc) and IBM SPSS version 28.0 (IBM).

## Results

Of the 83 patients analyzed, 22 were in the single group, 29 in the double group, and 32 in the quad group. A total of 846 epochs with adequate EGM quality as defined earlier were analyzed, resulting in 4230 EGMs when considering the 5 bipolar EGMs per catheter. Exemplary EGMs are presented in Supplemental Figure 1. End point verification was performed using 243 epochs and 1215 EGMs from the quad group. Baseline data of the patients show no difference between the groups except for hypercholesterolemia (Table 1).

**Table 1:** Baseline characteristics of patients

Parameters	All (n = 83)	Single (n = 22)	Double (n = 29)	Quadruple (n = 32)	*P* value
Age (y)	65 ± 10	64 ± 9	65 ±S 10	65 ± 11	.867
Women	28 (34)	9 (41)	12 (41)	7 (22)	.194
BMI (kg/m^2^)	27 ± 4	26 ± 4	27 ± 5	27 ± 4	.873
PAF	63 (76)	18 (82)	24 (83)	21 (66)	.222
CHA_2_DS_2_-VA score	2 (1–3)	2 (1–3)	1 (1–2)	2 (1–3)	.492
HTN	46 (55)	12 (55)	13 (45)	21 (66)	.263
CAD	8 (10)	3 (14)	1 (3)	4 (13)	.371
Hypercholesterolemia	30 (36)	5 (23)	8 (28)	17 (53)	.026
Diabetes	13 (16)	4 (18)	5 (17)	4 (13)	.818
LVEF (%)	59 ± 9	62 ± 8	60 ± 8	57 ± 9	.113
PLAX (mm)	40 ± 8	40 ± 7	38 ± 8	41 ± 8	.443
LAVI (mL/m^2^)	38 ± 14	40 ± 14	36 ± 12	39 ± 15	.617

Values are reported as mean ± standard deviation or median (interquartile range) for continuous and number (percentage) for categorical variables.

BMI = body mass index; CAD = coronary artery disease; HTN = hypertension; LAVI = left atrial volume indexed; LVEF = left ventricular ejection fraction; PAF = paroxysmal atrial fibrillation; PLAX = parasternal long axis.

### EGM feature extractions

The extracted features for the 3 groups are presented in Table 2. The HF power is reported for 3 different high-pass cutoff values at 50 Hz (HF50), 75 Hz (HF75), and 125 Hz (HF125). All HF features and BVA showed a significant reduction after the first application (*P* < .001). After the specified set of applications in the 3 groups (after), there is no difference in BVA (*P* = .614), HF50 (*P* = .500), HF75 (*P* = .434), and HF125 (*P* = .579) between the groups. However, after a 60-second waiting period, the groups were significantly different (*P* < .001 for BVA, *P* = .006 for HF50, *P* < .001 for HF75, and *P* = .028 for HF125). Although no differences were observed between the single and double groups, the quad group was significantly different from both the single and double groups in terms of voltage and all frequency-based features (*P* < .05, for all).

**Table 2:** EGM characteristics before and after PFA

Parameters		Before		Between		After		60 s	
Single (n = 68)									
BVA	(mV)	2.65	(1.37–7.40)			0.16	(0.09–0.44)	0.27	(0.16–0.72)
HF50	(μV^2^)	6776	(5690–32,763)			11	(9–63)	39	(31–174)
HF75	(μV^2^)	2968	(2565–14,570)			5	(4–21)	10	(8–54)
HF125	(μV^2^)	617	(499–3074)			1	(1–4)	3	(2–9)
Double (n = 64)									
BVA	(mV)	2.00	(1.28–5.30)			0.11	(0.09–0.57)	0.26	(0.12–0.72)
HF50	(μV^2^)	4128	(3723–17,711)			17	(11–83)	35	(22–135)
HF75	(μV^2^)	2037	(1836–8394)			6	(4–22)	13	(9–48)
HF125	(μV^2^)	4'70	(432–1514)			1	(1–5)	2	(1–8)
Quad (n = 93)									
BVA	(mV)	1.22	(0.63–2.91)	0.11	(0.06–0.17)	0.13	(0.06–0.41)	0.13	(0.07–0.32)
HF50	(μV^2^)	1967	(487–14,468)	8	(2–21)	11	(8–55)	8	(5–27)
HF75	(μV^2^)	1044	(212–4718)	3	(1–8)	5	(3–20)	3	(2–10)
HF125	(μV^2^)	197	(36, 983)	1	(0–1)	1	(0–3)	1	(0–2)

Values are reported as median (interquartile range).

BVA = bipolar voltage amplitude; EGM = electrogram; HF50, HF75, and HF125 = power of high-frequency component above 50 Hz, 75 Hz, and 125 Hz, respectively; n = number of analyzed applications; PFA = pulsed field ablation.

**Figure 2:**
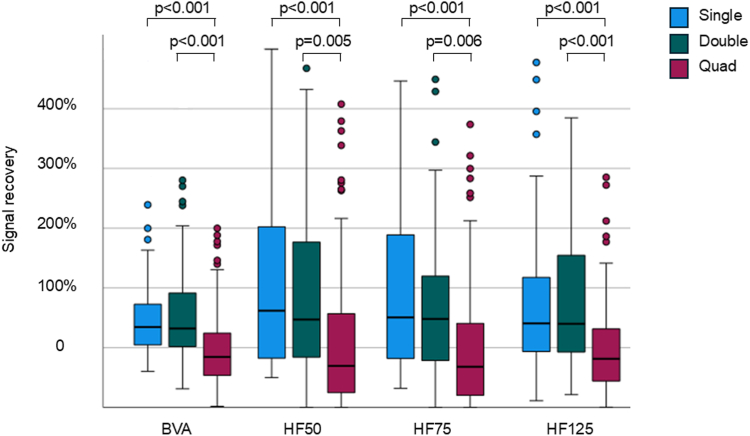
Observed median signal recovery during the 60-second waiting for the 4 investigated features: BVA and the absolute power of the high frequency of the EGM above 50 Hz (HF50), 75 Hz (HF75), and 125 Hz (HF125). BVA = bipolar voltage amplitude; EGM = electrogram.

The recovery of EGM features during the 60-second waiting period is presented in Figure 2. No differences were observed between single and double groups, but the quad group was significantly different in signal recovery from both the single and double groups.

Analysis of the distribution of the features based on the IQR showed a significant difference between the quad group and both the single and double groups for all features after a 60-second waiting period (*P* < .001).

### Acute efficacy based on pace capture

Overall, electrode pace capture in the quad group before the first application, between, and 60 seconds after the last ablation was possible in 87%, 23%, and 3% for the left superior PV; 51%, 4%, and 0% for the left inferior PV; 91%, 21%, and 5% for the right superior PV; and 71%, 12%, and 1% for the right inferior PV, respectively. A subanalysis for the 10 V and 5 V pacing is presented in Table 3. A significantly lower number of pace captures for the 5 V than 10 V pacing were observed for the left superior PV (*P* = .03) and right superior PV (*P* = .02) after the last ablation, which were both the first ablated PVs of each ipsilateral side of the LA. Pace-capture proportion was higher between the 2 ablation sets (between) for the 10 V pacing output than 5 V.

**Table 3:** Values of pace capture in the quad group

Pacing output	PV	Before	Between	60 s
10 V/1 ms	LSPV	92 (97/105)∗	33 (33/100)	4 (4/105)
	LIPV	51 (54/105)	5 (5/95)	0 (0/100)
	RSPV	95 (100/105)∗	31 (28/90)	3 (3/95)
	RIPV	71 (64/90)	13 (10/75)	1 (1/80)
5 V/1 ms	LSPV	76 (42/55)∗	4 (2/55)	0 (0/55)
	LIPV	49 (27/55)	4 (2/55)	0 (0/55)
	RSPV	84 (46/55)∗	2 (1/50)	7 (4/55)
	RIPV	71 (39/55)	9 (5/55)	0 (0/55)

Values are reported as % (captured electrodes/all paced electrodes).

LIPV = left inferior pulmonary vein; LSPV = left superior pulmonary vein; PV = pulmonary vein; RIPV = right inferior pulmonary vein; RSPV = right superior pulmonary vein.

∗ *P* < .05.

**Figure 3:**
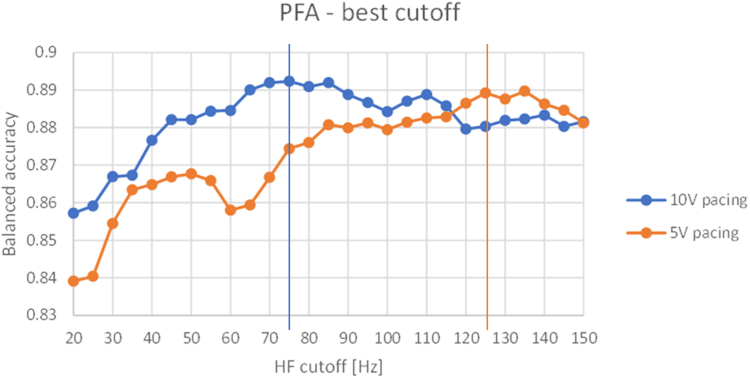
Plot of the balanced accuracy over different HF cutoffs for the end point of pace-capture prediction based on a 10 V (*blue*) and 5 V pacing (*orange*). HF = high frequency; PFA = pulsed field ablation.

The BA evaluated for different high-pass cutoffs from 20 to 150 Hz in steps of 5 Hz is presented in Figure 3. To predict exit block based on the lack of local pace capture, a cutoff frequency of 75 Hz (HF75) yielded the best performance for 10 V pacing, whereas a higher cutoff of 125 Hz (HF125) was optimal for 5 V pacing. In particular, with 10 V pacing, the feature HF75 achieved a BA of 0.892 at an optimal threshold of 31 μV^2^ (sensitivity 0.872; specificity 0.913). Similarly, for 5 V pacing, HF125 reached a BA of 0.890 at a threshold of 2.5 μV^2^ (sensitivity 0.898; specificity 0.882).

In both pacing conditions, prediction based on HF power slightly outperformed BVA-based prediction. For 10 V pacing, the optimal amplitude threshold was 0.41 mV (BA 0.871), whereas, for 5 V pacing, a threshold of 0.28 mV resulted in a BA of 0.852.

## Discussion

We performed a detailed, comparative analysis of how EGM features acutely change over incremental PFA applications. Our main observations are as follows: (1) a significant reduction was observed for all EGM features with the first PFA application. Increasing the number of PFA applications did not further reduce the values of the investigated features. (2) After a 60-second waiting period, recovery of the EGM features was observed in the single- and double-application groups, but not in the quadruple group. (3) Prediction of local pace capture using a 5 V/1 ms pulse assessed using EGM features achieved a maximal BA of 0.89 using HF125 with a 2.5 μV^2^ cutoff or a BVA of 0.28 mV. Our results provide the foundation for an intraprocedural end point to assess successful PFA lesion formation beyond stunning.

Unlike cryogenic or RF technologies, extensive heating or cooling of the tissue is not intended with PFA technology. Therefore, established end points such as tip temperature or impedance change with tissue heating are not applicable.^7^ To assess PFA lesion quality, sophisticated technologies such as optical coherence technology embedded in special catheters have been presented.^8^ However, simple EGM characterization strategies for the widely used pentaspline catheter are currently not available. As of now, immediate disappearance or attenuation of EGM signals and especially the BVA is commonly used as a simple end point to indicate acute electrical isolation. However, studies caution that EGM silencing alone may not guarantee adequate lesion depth or long-term success and effective PFA is more related to the biophysics of PFA than to mere EGM attenuation.^9^

### Impact of incremental ablations

The need for repetitive PFA application (stacking) for reliable PVI using the pentaspline catheter has been stated by the company of the pentaspline device based on the numerous pivotal trials.^10^ This stacking strategy is supported by animal studies using other catheter designs.^9^^,^^11^ However, the exact rationale for using quadruple applications with the pentaspline PFA catheter has not been published. In our ablation study, persistent lesion depletion over 60 seconds could only be achieved with 2 double applications, with a catheter rotation in between. In addition to the observed persistent lower HF and BVA values, the observed reduction of the confidence interval confirms that repetitive application with additional catheter rotation, as performed in the quad group, improves acute lesion homogeneity and consequently efficacy during the 60-second follow-up.

Our observations on BVA reduction are in line with other studies showing that postablation mapping at the PV ostium resulted in a BVA of <0.1 mV.^10^^,^^12^ The BVA of the pentaspline catheter was in the same range for all groups measured immediately after the intended number of applications. Importantly, BVA remained constant over 60 seconds in the quad group, but increased after single or double applications with 0.27 and 0.26 mV, respectively. A dynamic recovery of unipolar EGMs was also observed after 1 application in an animal model with a focal catheter over a period of 60 minutes.^13^

Although the IQR was not different for the BVA, differences in the distribution of the recovery of the frequency features (HF) could be observed for the different groups and different high-pass cutoffs, as displayed in Figure 2. The IQR was significantly smaller in the quad group than the single and double groups, potentially reflecting a more homogeneous cell destruction by the additional lesion and the repositioning of the catheter. Furthermore, given that the distribution of signal recovery of the HF features was smaller for higher-frequency cutoffs (HF125) than for lower ones (HF50), far-field signals might recover more than near-field ones, reflecting the importance of catheter field strength on the ablation efficacy.^14^

Regardless of the acute differences in local BVA observed with varying numbers of applications, the 12-month clinical outcome did not differ between 2 or 4 applications in a basket configuration in patients with paroxysmal AF.^15^ Whether this observation can be explained by the additional lesion set of 2 or 4 flower application requires further clarification.

### End point definition

The primary procedural end point for PVI is the acute electrical PVI confirmed by entrance and exit block.^16^ In direct comparison with pre- and postprocedural electroanatomic mapping using a multipolar high-density catheter, the accuracy of EGM- and pacing-based PVI assessment with the pentaspline catheter was reported as 91%.^17^ Lowering the pacing output from 10 to 5 V increased the accuracy of PVI detection to 97%. This effect was hypothesized to result from a more selective near-field capture at the lower electric field of 5 V, which is in line with our observation. For the first ablated ipsilateral PVs, we observed a significantly higher percentage of pace-captured electrode pairs for 10 V than 5 V. However, for the second ipsilateral vein, no difference was observed, most likely owing to the impact of overlapping lesions with the previously ablated ipsilateral PV. Furthermore, between the sets of 2 applications in the quad group, a higher percentage of pace capturing was observed for the high threshold (10 V) than the 5 V pacing. This difference diminished after the second block of double applications, most likely owing to the wider lesion set created with the 4 applications.

As reported previously,^18^^,^^19^ the HF components of the electrocardiogram represent near-field and lower-frequency far-field components. Although the low-frequency component of the electrocardiogram (HF50) decreased further with the second set of applications as observed in the quad group, the higher-frequency component (HF75 and HF125) remained constant. In conclusion, the above-described pace-capture difference between high (10 V) and lower output (5 V) is in line with the frequency-based EGM signal characteristics. Furthermore, BVA is also more influenced by the far-field signal and decreases with an additional set of ablations.

### Clinical application and interpretation

Although 1, 2, or 4 PFA applications resulted in comparable immediate reduction in all investigated features, only the set of 4 applications in basket configuration led to stable EGM features in both near-field and far-field features over the 60-second follow-up period. In contrast, the EGM features in the single- and double-application group showed significant recovery over the same period. For the end point definition of local pace capture based on a selective 5 V amplitude, an HF125 threshold of 2.5 μV^2^ results in the highest sensitivity and specificity. Given that frequency analysis is currently only available in 1 mapping system,^20^^,^^21^ a BVA threshold of 0.28 mV results in the most reliable discrimination of potential pace capture with 5 V after 4 applications. Besides the number of applications, no acute predictor of persistent EGM feature characteristics over the 60 seconds could be identified.

Whether recently published observations on the value of unipolar electrograms for lesion and contact assessment based on the visible “injury current” with clear unipolar voltage increase can improve PVI needs further investigation.^3^^,^^22^

### Limitations

Despite the analysis of more than 4200 EGMs, this remains a relatively small single-center study investigating focused on the impact of basket applications. Although animal models using different PFA catheter types have demonstrated temporal signal changes extending up to 20 minutes after application,^3^ our clinical data only report features at 60 seconds after the final application. However, a trend for signal recovery was observed after 1 or 2 applications even during this shorter period, confirming the value of repeated PFA applications. Furthermore, given that structured remapping was not performed, the association between the features and long-term persistence of PVI cannot be addressed in this study. Finally, our findings are specific to the investigated device. Whether other available circular catheters exhibit similar signal dynamics warrants further investigation.

## Conclusion

Despite widespread clinical use, the effect of repetitive PFA ablation on the persistence of cellular depolarization using the pentaspline ablation catheter remains incompletely understood. We observed that, despite comparable immediate effects on the observed BVA and the HF component of the overall signal, significant recovery occurred for single and double applications over 60 seconds. These findings underscore the need for 4 basket applications in vivo. For acute end point definition of PVI, an HF125 cutoff with a threshold of 2.5 μV^2^ or a BVA of 0.28 mV of all EGMs from the PV may serve as specific electrophysiological end points to confirm individual PVI instead of pacing. Whether this strategy translates into improved clinical outcomes or applies to other multielectrode ablation catheters warrants further investigation.

## Disclosures

P.B. has received research funding from the “University of Basel,” the “Stiftung für Herzschrittmacher und Elektrophysiologie,” the “Freiwillige Akademische Gesellschaft Basel,” the “Swiss Heart Foundation,” and Johnson & Johnson and reports personal fees from Bristol Myers Squibb (BMS), Boston Scientific, and Abbott, all outside the submitted work. P.K. reports speaker fees from BMS/Pfizer and grants from the Swiss National Science Foundation, Swiss Heart Foundation, Foundation for Cardiovascular Research Basel, and Machaon Foundation. S.K. has received funding from the “Swiss Heart Foundation,” outside of the submitted work. F.M. has been supported by Deutsche Forschungsgemeinschaft (SFB TRR219, project ID 322900939) and Deutsche Herzstiftung. Until May 2024, F.M. has received speaker honoraria/consulting fees from Ablative Solutions, AstraZeneca, Inari, Medtronic, Merck, Novartis, Philips, and ReCor Medical, all outside the submitted work. C.S. is a member of Medtronic Advisory Board Europe and Boston Scientific Advisory Board Europe and received educational grants from Biosense Webster and Biotronik, a research grant from the European Union’s FP7 program and Biosense Webster, and lecture and consulting fees from Abbott, Medtronic, Biosense Webster, Boston Scientific, MicroPort, and Biotronik, all outside the submitted work. M.K. reports grants from the Swiss National Science Foundation (grant numbers 33CS30_148474, 33CS30_177520, 32473B_176178, 32003B_197524), the Swiss Heart Foundation, the Foundation for Cardiovascular Research Basel, the University of Basel, Bayer, Pfizer, Boston Scientific, BMS, and Biotronik and grants and personal fees from Daiichi Sankyo, all outside the submitted work. B.S. reports receiving a travel grant from Boston Scientific, outside the submitted work. Others have nothing to declare.

## Footnotes

Appendix Supplementary data associated with this article can be found in the online version at https://doi.org/10.1016/j.hroo.2026.01.019.
