# Supplementary material for: Single or multiple pulsed field applications: Intracardiac electrogram changes and implications for procedural end points
Source: Heart Rhythm O2. 2026 Jan 27;7(4):649–55. doi: 10.1016/j.hroo.2026.01.019 (PMC13107057; doi:10.1016/j.hroo.2026.01.019)
Supplement: Supplementary Figure [file mmc1.docx]

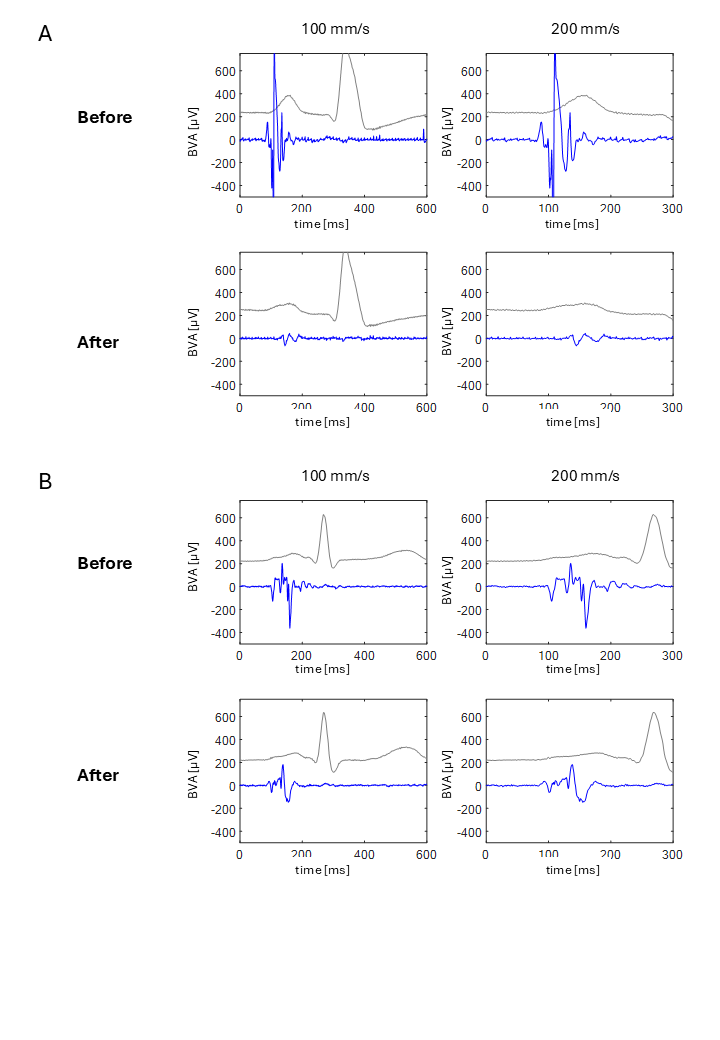


Supplemental figure: Representative examples of intracardiac electrograms (EGMs) recorded before and after ablation. A: Successful ablation, with complete elimination of the near-field signal and bipolar voltage amplitude (BVA). B: In contrast, the second example shows persistent near-field activity post-ablation, with the HF125 feature remaining above the defined threshold. The surface ECG, displayed in grey, was used for time-window synchronization.
